# Akt2 ablation prolongs life span and improves myocardial contractile function with adaptive cardiac remodeling: role of Sirt1‐mediated autophagy regulation

**DOI:** 10.1111/acel.12616

**Published:** 2017-07-05

**Authors:** Jun Ren, Lifang Yang, Li Zhu, Xihui Xu, Asli F. Ceylan, Wei Guo, Jian Yang, Yingmei Zhang

**Affiliations:** ^1^ Department of Cardiology and Shanghai Institute of Cardiovascular Diseases Zhongshan Hospital Fudan University Shanghai 200032 China; ^2^ Center for Cardiovascular Research and Integrative Medicine School of Pharmacy University of Wyoming Laramie WY 82071 USA; ^3^ Department of Anesthesiology Xi'an Children Hospital Xi'an 710003 China; ^4^ Department of Animal Sciences University of Wyoming Laramie WY 82071 USA; ^5^ Department of Cardiovascular Surgery Xijing Hospital Fourth Military Medical University Xi'an 710032 China

**Keywords:** aging, Akt, autophagy, cardiac geometry, contractile function

## Abstract

Aging is accompanied with unfavorable geometric and functional changes in the heart involving dysregulation of Akt and autophagy. This study examined the impact of Akt2 ablation on life span and cardiac aging as well as the mechanisms involved with a focus on autophagy and mitochondrial integrity. Cardiac geometry, contractile, and intracellular Ca^2+^ properties were evaluated using echocardiography, IonOptix^®^ edge‐detection and fura‐2 techniques. Levels of Sirt1, mitochondrial integrity, autophagy, and mitophagy markers were evaluated using Western blot. Our results revealed that Akt2 ablation prolonged life span (by 9.1%) and alleviated aging (24 months)‐induced unfavorable changes in myocardial function and intracellular Ca^2+^ handling (SERCA2a oxidation) albeit with more pronounced cardiac hypertrophy (58.1%, 47.8%, and 14.5% rises in heart weight, wall thickness, and cardiomyocyte cross‐sectional area). Aging downregulated levels of Sirt1, increased phosphorylation of Akt, and the nuclear transcriptional factor Foxo1, as well as facilitated acetylation of Foxo1, the effects of which (except Sirt1 and Foxo1 acetylation) were significantly attenuated or negated by Akt2 ablation. Advanced aging disturbed autophagy, mitophagy, and mitochondrial integrity as evidenced by increased p62, decreased levels of beclin‐1, Atg7, LC3B, BNIP3, PTEN‐induced putative kinase 1 (PINK1), Parkin, UCP‐2, PGC‐1α, and aconitase activity, the effects of which were reversed by Akt2 ablation. Aging‐induced cardiomyocyte contractile dysfunction and loss of mitophagy were improved by rapamycin and the Sirt1 activator SRT1720. Activation of Akt using insulin or Parkin deficiency prevented SRT1720‐induced beneficial effects against aging. In conclusion, our data indicate that Akt2 ablation protects against cardiac aging through restored Foxo1‐related autophagy and mitochondrial integrity.

## Introduction

Biological aging is an irreversible process featured by progressive myocardial remodeling, reduced cardiac reserve, diastolic dysfunction, and a higher cardiac morbidity and mortality in the elderly (Lakatta, 1999; Sussman & Anversa, 2004; Yang *et al*., 2005; Shimizu & Minamino, 2016). A number of hypotheses have been postulated for cardiac aging including oxidative stress, depletion of cellular fuel, myosin heavy chain isozyme switch, apoptosis, mitochondrial injury, autophagy dysregulation, and intracellular Ca^2+^ mishandling (Lakatta, 1999; Yang *et al*., 2005, 2006; Boengler *et al*., 2007; Feridooni *et al*., 2015; Horn, 2015). Nonetheless, the precise machineries behind cardiac aging still remain somewhat elusive. Recent evidence from our laboratory and others has depicted a unique role for phosphoinositide 3‐kinase (PI3K) and its downstream‐signaling target protein kinase B (Akt) in aging‐induced pathological changes in the heart (Inuzuka *et al*., 2009; Hua *et al*., 2011; Pillai *et al*., 2014). It was shown that the on‐and‐off switching of the PI3K/Akt pathway, particularly by insulin and insulin‐like growth factor‐1 (IGF‐1), serves as a powerful physiological integrator rudimentary to life span and aging (Nair & Ren, 2012; O'Neill, 2013). Our data have revealed an essential role for diminished autophagy, an evolutionarily conserved lysosome‐dependent process for turnover of proteins and organelles, in Akt overactivation‐induced accentuation of cardiac aging process (Hua *et al*., 2011). Autophagy plays a key role for biological aging process and cardiac homeostasis. Diminished autophagy has been shown to accelerate mammalian aging (Hars *et al*., 2007; Simonsen *et al*., 2008; Toth *et al*., 2008; Alvers *et al*., 2009; Taneike *et al*., 2010), in association with accumulation of damaged intracellular components including protein aggregate (Cuervo *et al*., 2005; Zhang & Cuervo, 2008; Gottlieb *et al*., 2009; Taneike *et al*., 2010). Moreover, defective autophagy facilitates ventricular remodeling, contractile defects, and heart failure (Nakai *et al*., 2007; Yitzhaki *et al*., 2009). Given the critical role for Akt in the regulation of cardiac survival and life span (Hay & Sonenberg, 2004; Hahn‐Windgassen *et al*., 2005), this study was designed to examine the role of Akt2 ablation on aging‐induced geometric, functional, and intracellular Ca^2+^ homeostatic changes in the heart, with a focus on autophagy and mitochondrial integrity. The Akt downstream‐signaling molecules including Forkhead transcriptional factors of Forkhead box O class (Foxo) were examined in wild‐type and Akt2 knockout mice. FOXOs in particular Foxo1 are associated with life span and serve as a paradigm for the understanding of aging‐related chronic diseases such as cancer and diabetes (Charitou & Burgering, 2013). Levels of autophagy and mitophagy protein markers including phosphatase and tensin homolog (PTEN)‐induced kinase 1 (PINK1), Parkin, and BCL2/adenovirus E1B 19‐kDa‐interacting protein 3 (BNIP3) were examined in young or aged mouse hearts. It has been depicted that mitochondrial priming is mediated either by the PINK1‐Parkin signaling pathway or the mitophagic receptors Nix and BNIP3 (Ding & Yin, 2012), in an effort to eliminate damaged mitochondria and to preserve mitochondrial integrity. Our findings indicated that Akt2 ablation prolongs life span and improves myocardial contractile function with a possible adaptive cardiac remodeling through the Sirt1‐mediated autophagy regulation.

## Results

### General biometric and echocardiographic characteristics

**Figure 1:**
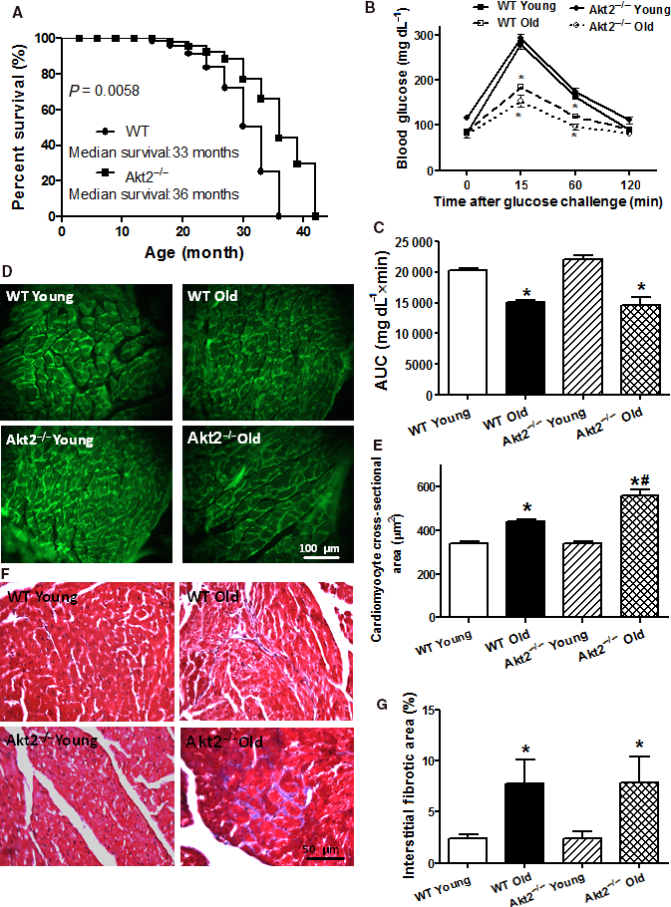
Cumulative survival curve, intraperitoneal glucose tolerance test (IPGTT, 2 g kg^−1^), area underneath curve (AUC), and myocardial morphology in male WT and Akt2 knockout (Akt2^−/−^) mice at young (3–4 months) and old (24–26 months) ages. (A) Kaplan–Meier cumulative survival rate plotted against age in months in WT and Akt2^−/−^ mice. The log‐rank test was performed to compare the two curves (*P* = 0.0058). (B) Plasma glucose levels within 120 min following IPGTT challenge; (C) AUC; (D) Representative FITC‐conjugated lectin staining depicting the transverse sections of left ventricular myocardium (×400); (E) Quantitative analysis of cardiomyocyte cross‐sectional area; (F) Representative Masson trichrome staining depicting interstitial fibrosis; and (G) Quantitative analysis of fibrotic area (Masson's trichrome‐stained area in light blue color normalized to the total myocardial area). Mean ± SEM,* n* = 31 mice per group for panel A; 6–7 mice per group for panel B–C; 19–20 fields from five mice per group for panel D–E; and 9–10 fields from five mice per group for panel F–G, *P < 0.05 vs. respective WT young, ^#^ *P* < 0.05 vs. WT old group.

The biometric profiles of young or old WT and Akt2 knockout mice are summarized in Table 1. Akt2 ablation did not elicit notable effects on body and organ weights compared with WT mice. Advanced aging prompted greater body (19.0%) and organ (27.9% in heart, 25.5% in kidney, and 55.8% in spleen but not liver) weights as well as heart size (30.1% rise, normalized to tibial length) (*P* < 0.05). Akt2 ablation accentuated aging‐induced cardiac hypertrophy (58.2% in heart weight, 57.7% in heart‐to‐body weight, and 52.1% in heart‐to‐tibial length ratios compared to old WT mice) and hepatomegaly (20.1% rise compared to young WT mice) without affecting aging‐induced responses on kidney and spleen (organ weights or sizes). Neither advanced aging nor Akt2 ablation, or both, overtly altered blood glucose levels. Aging, but not Akt2 ablation, increased plasma insulin and triglyceride levels (*P* < 0.05), the effect of which was unaffected by Akt2 ablation (Table 1). The Kaplan–Meier curve comparison depicts that Akt2 knockout mice display significantly better survival (9.1%) than WT mice. The median survivals were 33 and 36 months for WT and Akt2^−/−^ mice, respectively (*P* = 0.0058, Fig. 1A). Aging, but not Akt2 ablation, significantly increased left ventricular mass, end‐diastolic diameter (EDD), and end‐systolic diameter (ESD) along with reduced fractional shortening (*P* < 0.05), the effect of which was reversed by Akt2 ablation with the exception of left ventricular mass (where a more pronounced increased was observed, *P* < 0.001). Neither aging nor Akt2 ablation, or both, significantly affected heart rate, wall thickness except that the combination of the two overtly increased left ventricular wall thickness (47.8% rise compared with old WT mice), consistent with an elevated left ventricular mass (17.4% rise compared with old WT mice) in aged Akt2^−/−^ mice (Table 1).

**Table 1:** Biometric and echocardiographic parameters of WT and Akt2^−/−^ mice at young and old ages

Parameter	WT Young	WT Old	Akt2^−/−^ Young	Akt2^−/−^ Old
Body weight (g)	24.7 ± 0.6	29.4 ± 0.4*	24.4 ± 0.4	29.5 ± 1.0*
Heart weight (mg)	129 ± 3	165 ± 3*	126 ± 2	261 ± 15*,#
Heart/Body weight (mg g^−1^)	5.23 ± 0.17	5.63 ± 0.10	5.16 ± 0.05	8.88 ± 0.54*,#
Tibial length (mm)	20.9 ± 0.5	20.7 ± 0.4	20.3 ± 0.4	21.3 ± 0.5
Heart/Tibial length (mg mm^−1^)	6.18 ± 0.15	8.04 ± 0.24*	6.21 ± 0.20	12.23 ± 0.58*,#
Liver weight (g)	1.35 ± 0.04	1.49 ± 0.18	1.29 ± 0.02	1.60 ± 0.07*
Liver/Body weight (mg g^−1^)	54.7 ± 1.1	50.7 ± 0.99	53.3 ± 1.0	54.1 ± 2.0
Kidney weight (mg)	302 ± 7	379 ± 6*	299 ± 4	366 ± 10*
Kidney/Body weight (mg g^−1^)	12.3 ± 0.2	12.9 ± 0.3	12.3 ± 0.2	12.5 ± 0.4
Spleen weight (mg)	104 ± 5	162 ± 7*	103 ± 3	183 ± 12*
Spleen/Body weight (mg g^−1^)	4.03 ± 0.29	5.52 ± 0.27*	4.27 ± 0.22	6.24 ± 0.44*
Fasting plasma BG (mg dL^−1^)	104 ± 5	106 ± 4	103 ± 5	108 ± 5
Plasma insulin (ng mL^−1^)	0.085 ± 0.011	0.249 ± 0.032*	0.070 ± 0.012	0.277 ± 0.018*
Plasma triglycerides (mg dL^−1^)	55.3 ± 5.5	82.1 ± 5.5*	57.1 ± 8.4	80.6 ± 5.6*
Heart rate (bpm)	500 ± 19	483 ± 12	478 ± 8	453 ± 17
Wall thickness (mm)	1.22 ± 0.07	1.13 ± 0.07	1.07 ± 0.02	1.67 ± 0.08*,#
EDD (mm)	2.69 ± 0.13	3.58 ± 0.09*	2.71 ± 0.05	2.75 ± 0.14#
ESD (mm)	1.41 ± 0.04	2.35 ± 0.13*	1.49 ± 0.04	1.32 ± 0.06#
Fractional shortening (%)	50.6 ± 3.6	35.4 ± 2.8*	45.1 ± 0.8	52.6 ± 1.6#
Left ventricular mass (mg)	92.0 ± 6.2	116.0 ± 7.2*	87.7 ± 3.0	136.2 ± 7.9*,#

BG = blood glucose; EDD = end‐diastolic diameter; ESD = end‐systolic diameter; Mean ± SEM, *n* = 8 mice per group, *P < 0.05 vs. WT young group, ^#^ *P* < 0.05 vs. WT old group.

### IPGTT and myocardial histology in young or old WT and Akt2 knockout mice

Following acute intraperitoneal glucose challenge, changes in plasma glucose levels of the young WT and Akt2^−/−^ mice displayed similar patterns, that is, peaking at 15 min and returning to near baseline after 120 min. However, the postchallenge glucose levels achieved significantly lower levels between 15 and 60 min in both aged groups (*P* < 0.001), indicating poor glucose capacity. The basal fasting plasma glucose levels were comparable among all groups (*P* > 0.05), excluding the existence of full‐blown diabetes (Fig. 1B). These findings were supported by the area under the IPGTT curve (AUC) where diminished AUC levels were observed in aged groups (*P* < 0.001) with little effect of Akt2 ablation at the young age (Fig. 1C). To assess the impact of Akt2 ablation on cardiac remodeling in aging, myocardial histology including cardiomyocyte cross section and interstitial fibrosis was examined. Lectin staining revealed an increased cardiomyocyte transverse cross‐sectional area in aged mouse hearts (*P* < 0.001), with a more pronounced increase in the aged Akt2^−/−^ mice (*P* < 0.001), consistent with increased LV mass and heart weights in aged mice. Little effect was noted in cardiomyocyte cross‐sectioning from Akt2 ablation itself (Fig. 1D,E). Masson's trichrome staining revealed that aging enhanced interstitial fibrosis (*P* = 0.0496), the effect of which was unaffected by Akt2 ablation with little effect from Akt2 ablation itself (Fig. 1F,G).

### Cardiomyocyte contractile, stimulation frequency‐PS relationship, and intracellular Ca^2+^ transient properties

**Figure 2:**
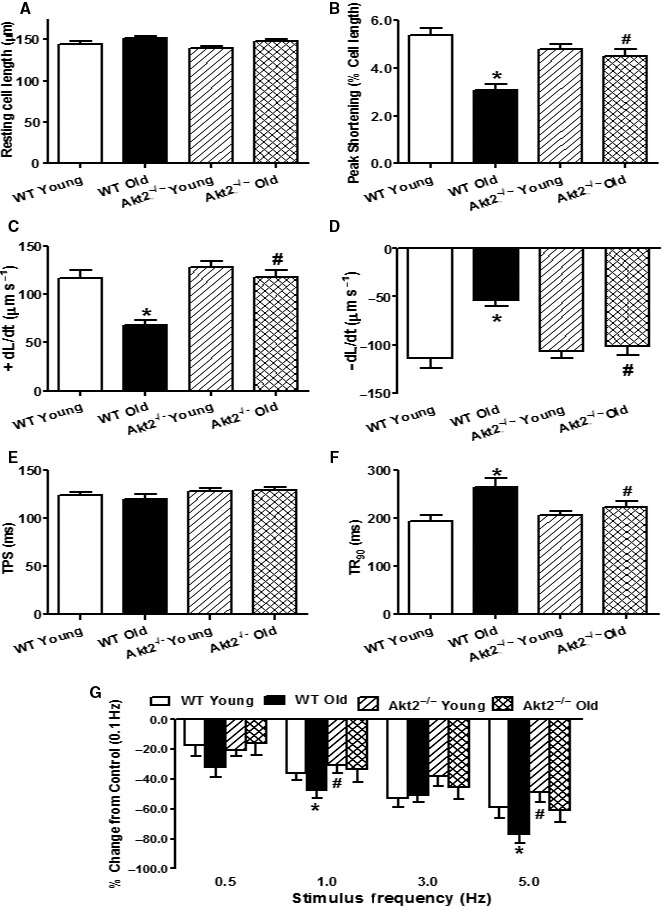
Cardiomyocyte mechanical function and frequency‐shortening response in male WT and Akt2 knockout (Akt2^−/−^) mice at young (3–4 months) and old (24–26 months) ages. (A) Resting cell length; (B) Peak shortening (PS, normalized to resting cell length); (C) Maximal velocity of shortening (+ dL/dt); (D) Maximal velocity of relengthening (− dL/dt); (E) Time‐to‐PS (TPS); (F) Time‐to‐90% relengthening (TR_90_); and (G) Changes in peak shortening amplitude of cardiomyocytes (normalized to that obtained at 0.1 Hz from the same cell) at various stimulus frequencies (0.1–5.0 Hz). Mean ± SEM,* n* = 105–111 cells (panel A–F) or 23–24 cells (Panel G) from five mice per group, *P < 0.05 vs. WT young group, ^#^ *P* < 0.05 vs. WT old group.

Neither advanced aging nor Akt2 ablation significantly affected resting cardiomyocyte length. Advanced aging significantly reduced peak shortening, maximal velocities of shortening/relengthening (± dL/dt) (all with *P* < 0.001) and prolonged time‐to‐90% relengthening (TR_90_, *P* = 0.0010) without affecting time‐to‐peak shortening (TPS). While Akt2 ablation itself did not elicit any significant effects on the cardiomyocyte mechanical parameters of young mice, it rescued aging‐induced alterations in PS, ± dL/dt (all with *P* < 0.001) and TR_90_ (*P* = 0.0010) without affecting TPS (Fig. 2A–F).

Mouse hearts normally contract at very high frequencies (> 500 bpm), whereas our recordings were performed at 0.5 Hz. To evaluate the impact of aging and/or Akt2 ablation on cardiac contractile function under higher frequencies, we increased stimulus frequency up to 5.0 Hz (300 beats min^−1^) and recorded steady‐state PS amplitude. Cardiomyocytes were initially stimulated to contract at 0.5 Hz for 5 min to ensure steady state prior to commencing the frequency response. Figure 2E displays a negative staircase of PS with increased frequency in all four groups with a steeper decline in the aged mice (*P* < 0.05). Although Akt2 ablation itself failed to elicit any obvious effects on the patterns of frequency‐shortening response, it attenuated aging‐induced decline in PS amplitude (*P* < 0.05). These data favor a possible role for improved intracellular Ca^2+^ cycling and stress intolerance in Akt2 ablation‐offered beneficial mechanical response in aging.

**Figure 3:**
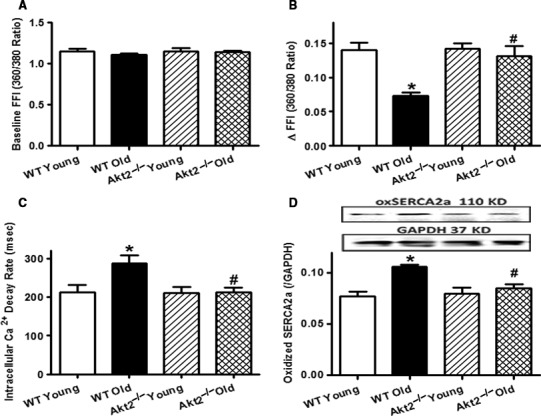
Intracellular Ca^2+^ transients and sarco(endo)plasmic reticulum Ca^2+^ ATPase (SERCA2a) oxidation (oxSERCA2a) in cardiomyocytes or hearts from young (3–4 months) or old (24–26 months) male WT and Akt2^−/−^ mice. (A) Baseline fura‐2 fluorescence intensity (FFI); (B) Electrically stimulated rise in FFI (ΔFFI); (C) Single exponential intracellular Ca^2+^ decay rate; and (D) Levels of oxidized SERCA2a. Inset: Representative gel blots depicting oxSERCA2a and loading control GAPDH. Mean ± SEM,* n* = 82 cells from five mice per group for panel A–C; *n* = 8 and 9 mice for WT and Akt2^−/−^ groups, respectively, for panel D, *P < 0.05 vs. WT young group, ^#^ *P* < 0.05 vs. WT old group.

To explore the possible mechanisms between aging and Akt2 ablation, intracellular Ca^2+^ handling was evaluated using fura‐2 fluorescence techniques. Our data revealed that neither aging nor Akt2 knockout, or both, significantly affected resting intracellular Ca^2+^ levels. Aging, but not Akt2 ablation, significantly decreased the electrically stimulated rise in intracellular Ca^2+^ (ΔFFI, *P* < 0.001) and intracellular Ca^2+^ clearance (*P* = 0.0019), the effects of which were reversed by Akt2 ablation. Last but least, advanced aging promoted oxidation of sarco(endo)plasmic reticulum Ca^2+^‐ATPase (SERCA2a, *P* = 0.0006), the effect of which was mitigated by Akt2 knockout with little effect by Akt2 knockout itself (Fig. 3).

### Effect of aging and Akt2 ablation on Sirt1, autophagy, mitophagy, and mitochondrial integrity

**Figure 4:**
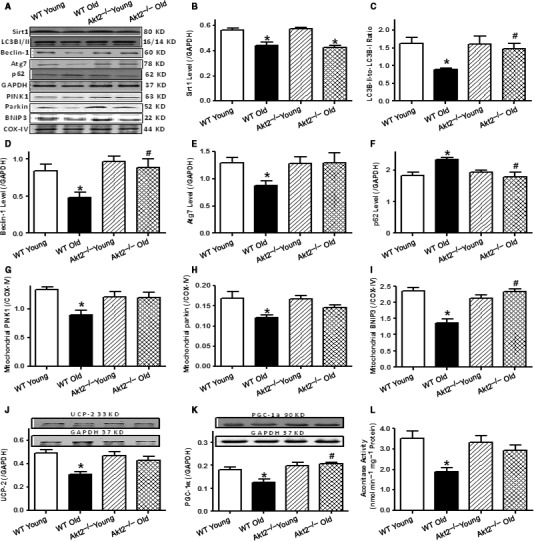
Levels of the longevity gene Sirt1, autophagy protein markers, mitophagy protein markers, mitochondrial proteins UCP‐2, PGC‐1α, and aconitase activity in myocardium from male young (3–4 months) or old (24–26 months) WT and Akt2^−/−^ mice. (A) Representative gel blots depicting levels of Sirt1, LC3BI/II, Atg7, p62, PINK1, Parkin, BNIP3, and respective loading controls (GAPDH and COX‐IV) using specific antibodies; (B) Sirt1 levels (*n* = 10, 9, 9, and 8 mice for the four animal groups shown from left to right); (C) LC3B‐II‐to‐LC3B‐I ratio (*n* = 16, 18, 13, and 16 mice from left to right); (D) Beclin‐1 levels (*n* = 7, 11, 7, and 8 mice from left to right); (E) Atg7 levels (*n* = 9, 9, 9, and 8 mice from left to right); (F) p62 levels (*n* = 8, 11, 9, and 8 mice from left to right); (G) Mitochondrial PINK1 levels (normalized to COX‐IV,* n* = 9, 10, 12, and 10 mice from left to right); (H) Mitochondrial Parkin levels (normalized to COX‐IV,* n* = 9, 10, 9, and 9 mice from left to right); (I) Mitochondrial BNIP3 levels (normalized to COX‐IV,* n* = 11, 8, 11, and 11 mice from left to right); (J) UCP‐2 levels (*n* = 6, 7, 5, and 5 mice from left to right); (K) PGC‐1α levels (*n* = 6, 7, 5, and 5 mice from left to right); Insets: Representative gel blots depicting levels of UCP‐2, PGC‐1α, and GAPDH using specific antibodies; and (L) Aconitase activity (*n* = 8 mice per group). Mean ± SEM, *P < 0.05 vs. WT young group, ^#^ *P* < 0.05 vs. WT old group.

To elucidate the mechanisms of action behind aging and/or Akt2 ablation‐induced cardiac mechanical and intracellular Ca^2+^ responses, longevity marker Sirt1, autophagy, mitophagy, and mitochondrial integrity were examined. Our data depicted that aging significantly downregulated Sirt1 levels in a comparable manner in WT and Akt2^−/−^ mice. Levels of autophagy proteins including LC3B, Beclin‐1, and Atg7 were downregulated along with elevated autophagosome adaptor protein p62 in aged WT mice (*P* < 0.05). Although Akt2 ablation itself did not affect these autophagy protein markers, it negated aging‐induced changes in autophagy. To further evaluate aging‐ and Akt2 ablation‐induced changes in mitochondrial autophagy and integrity, protein markers of mitophagy and mitochondrial integrity as well as mitochondrial aconitase activity were evaluated. Our data revealed that aging downregulated the levels of the mitophagy protein markers PINK1 (*P* = 0.0080), Parkin (*P* = 0.0096), and BNIP3 (*P* < 0.0001), as well as the mitochondrial proteins UCP‐2 (*P* = 0.0018) and PGC‐1α (*P* = 0.0006) along with reduced mitochondrial aconitase activity (*P* = 0.0025), the effects of which were effectively obliterated by Akt2 ablation. Akt2 knockout itself failed to affect the levels of these mitophagy or mitochondrial protein markers (Fig. 4). Our result shown in Fig. S1 (Supporting information) validated Akt2 ablation in Akt2^−/−^ mouse hearts with little effect on Akt2 levels by aging in WT mice.

### Effect of aging and Akt2 knockout on phosphorylation of Akt and Foxo1 as well as acetylation of Foxo1

**Figure 5:**
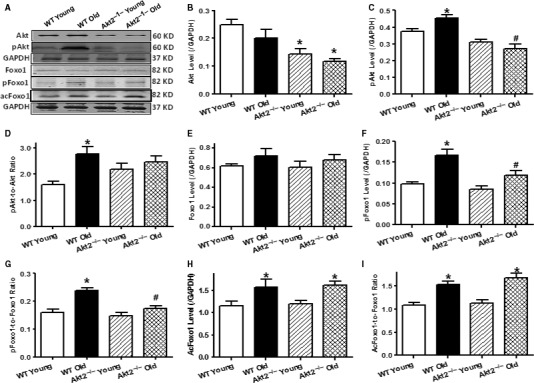
Levels of pan and phosphorylated (acetylated) Akt or Foxo1 in myocardium from male young (3–4 months) or old (24–26 months) WT and Akt2^−/−^ mice. (A) Representative gel blots depicting levels of Pan Akt, pan Foxo1, phosphorylated Akt (pAkt), phosphorylated Foxo1 (pFoxo1), acetylated Foxo1 (acFoxo1), and loading control GAPDH using specific antibodies; (B) Akt levels; (C) pAkt levels; (D) pAkt‐to‐Akt ratio (for panel B–D: *n* = 17, 17, 14, and 12 mice for four animal groups from left to right); (E) Foxo1 levels; (F) pFoxo1 levels; (G) pFoxo1‐to‐Foxo1 ratio; (H) acFoxo1 levels; and (I) acFoxo1‐to‐Foxo1 ratio (for panel E–I: *n* = 10 and 9 for WT and Akt2^−/−^ groups, respectively). Mean ± SEM, *P < 0.05 vs. WT young group, ^#^ *P* < 0.05 vs. WT old group.

To elucidate the cell signaling mechanisms involved in aging and/or Akt2 knockout‐induced changes in cardiac function, mitochondrial integrity, and autophagy, Western blotting was performed on signaling molecules including Akt and the Akt downstream transcriptional factor Forkhead box O‐1 (Foxo1). Advanced aging overtly activated Akt and Foxo1 without affecting the pan protein expression of Akt and Foxo1 (*P* < 0.0001), the effect of which was mitigated by Akt2 knockout. As expected, Akt2 ablation significantly decreased the pan protein levels of Akt (but not Foxo1, *P* = 0.0004) in a comparable manner in young and aged mice. Our further assessment revealed that advanced aging promoted Foxo1 acetylation (*P* < 0.0001), the effect of which was unaffected by Akt2 knockout. Akt2 knockout itself failed to alter acetylation of Foxo1 (Fig. 5).

### Effects of autophagy induction and Sirt1 activation on aging‐induced cardiomyocyte mitophagy and contractile responses

**Figure 6:**
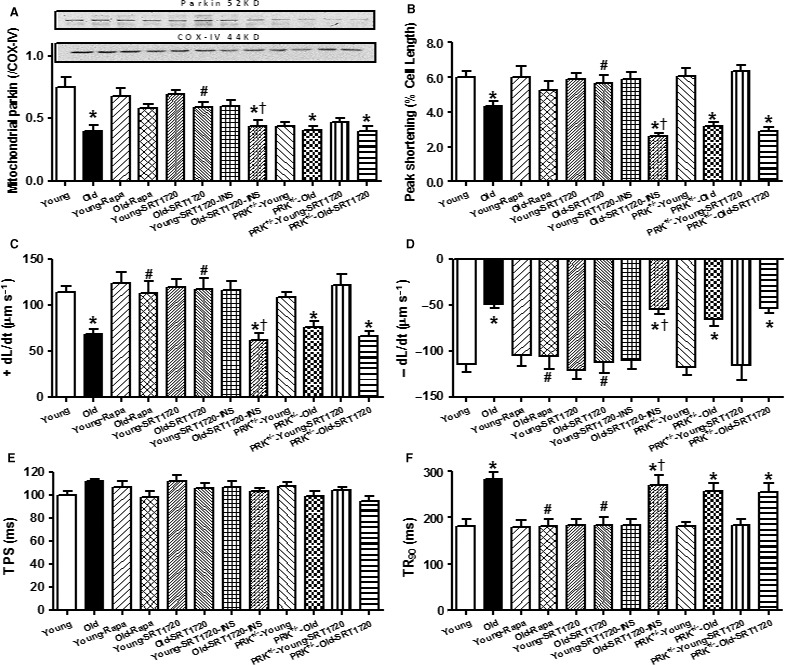
Effect of autophagy induction, Sirt1 activation, Akt activation, and Parkin inhibition on mitophagy marker Parkin and cardiomyocyte function in cardiomyocytes from young (4 months) and old (24 months) WT mice. Cardiomyocytes from young or old WT and Parkin heterozygous deletion (PRK ^−/−^) were incubated for 4 h with the autophagy inducer rapamycin (5 μm), the Sirt1 activator SRT1720 (1 μm) prior to the biochemical and mechanical assessment. A cohort of cardiomyocytes from young or old WT mouse cardiomyocytes was incubated with SRT1720 for 4 h in the absence or presence of the Akt activator insulin (100 nm). (A) Parkin expression; Inset: Representative gel blots depicting levels of mitochondrial fraction Parkin and COX‐IV (as the mitochondrial loading control); (B) Peak shortening (normalized to resting cell length); (C) Maximal velocity of shortening (+ dL/dt); (D) Maximal velocity of relengthening (− dL/dt); (E) Time‐to‐PS (TPS); and (F) Time‐to‐90% relengthening (TR_90_). Mean ± SEM,* n* = 8–9 isolations (panel A) or 61 cells from four mice (panel B–F), *P < 0.05 vs. respective young group, ^#^ *P* < 0.05 vs. old group, ^†^ *P* < 0.05 vs. corresponding insulin absent group.

To further examine the role of autophagy and Sirt1 in aging‐induced cardiac contractile alterations, cardiomyocytes from young and aged WT mice were incubated with the autophagy inducer rapamycin (5 μm) or the Sirt1 activator SRT1720 (1 μm) in the absence or presence of insulin (100 nm, to activate Akt). Our data revealed that both rapamycin and SRT1720 failed to elicit any mechanical or mitophagy (Parkin) responses in cardiomyocytes from young mice although both pharmacological agents alleviated or abrogated aging‐induced cardiomyocyte mitophagy and contractile responses including reduced mitochondrial Parkin levels, PS and ± dL/dt, and prolonged TR_90_ (with unchanged TPS) (*P* < 0.05). Interestingly, the SRT1720‐induced beneficial responses against aging were nullified by Akt activation using insulin with little notable effects from insulin in young cardiomyocytes. Furthermore, deficiency of Parkin using cardiomyocytes from the heterozygous Parkin knockout mouse model also nullified SRT1720‐offered beneficial effects against aging (mitophagy and contractile function) (*P* < 0.05 compared with the old‐SRT1720 group). Parkin deficiency failed to affect mitophagy and contractile function at young age nor did it elicit additive effects on aging‐induced induced responses on mitophagy and contractile function (Fig. 6) . These data support a beneficial role for autophagic induction, Sirt1 activation, and Parkin in cardiac mitophagy and functional homeostasis in aging. Last but not least, inhibition of autophagy using 3‐MA (3 mm, 4 h) significantly attenuated the beneficial effect of Akt2 ablation against aging in cardiomyocytes without eliciting any notable response at the young age (Fig. S2, Supporting information), suggesting that Akt2 ablation is cell autonomous dependent.

## Discussion

Our salient findings revealed that ablation of Akt2 prolonged life span and rescued against aging‐associated contractile and intracellular Ca^2+^ defects, despite more pronounced ‘adaptive’ cardiac hypertrophy. In addition, Akt2 ablation alleviated aging‐associated mitochondrial injury. Our data further depicted an essential role for the Akt‐Foxo1 phosphorylation and Sirt1‐Foxo1 deacetylation cascades in regulation of autophagy in cardiac aging (as depicted in Fig. S3, Supporting information). The permissive roles of autophagy and Sirt1 in Akt2 knockout‐offered beneficial action received consolidation from the observations that autophagy inhibition using 3‐MA negated Akt2 ablation‐offered beneficial response against cardiac aging, whereas autophagy induction using rapamycin or Sirt1 activation using SRT1720 alleviated aging‐induced cardiomyocyte mitophagy and mechanical derangements. The fact that SRT1720‐offered beneficial response in aging was nullified or attenuated by insulin suggests possible offsetting effects between Akt and Sirt1 in cardiac homeostasis in aging. Our finding that Parkin knockout obliterated SRT1720‐offered benefit against aging depicted a mitophagy‐mediated mechanism in Sirt1‐offered benefit in aging. These results collectively suggest a possible role for Akt2 (the major Akt isozyme in the heart) and autophagy in cardiac aging and more importantly, the therapeutic potential for autophagy or mitophagy in cardiac aging.

Cardiac aging is characterized by unfavorable cardiac remodeling and function including cardiac hypertrophy, interstitial fibrosis, compromised contractility, and prolonged diastolic duration (Kudo *et al*., 1995; Yang *et al*., 2005, 2006; Gottlieb *et al*., 2009). This is in line with cardiac hypertrophy, interstitial fibrosis, intracellular Ca^2+^ dysregulation, reduced Ca^2+^ cycling capacity, and compromised contractile function in aging hearts from our current study. To our surprise, Akt2 ablation negated aging‐induced cardiac contractile dysfunction with a more pronounced remodeling. More prominent changes in heart mass/size, and cardiomyocyte cross‐sectional area (but not fibrosis) were noted in aged Akt2^−/−^ mice, favoring an important role for Akt2 in aging as opposed to young hearts. Cardiac remodeling (hypertrophy and interstitial fibrosis) is common manifestation of aging, in transition from physiological to pathological hypertrophy, en route to the ultimate heart failure (Wei, 1992). With the improved cardiac function in aging, the more pronounced cardiac hypertrophy in the face of Akt2 ablation seemed to suggest a state of adaptive cardiac hypertrophy in aged Akt2^−/−^ hearts. Akt2 knockout did not elicit any notable cardiac geometric or morphological effect at young age, suggesting that ablation of Akt2 may take time to impose any notable cardiac remodeling and contractile effects. In cardiac aging, cardiomyocytes displayed decreased intracellular Ca^2+^ release in response to electrical stimulus and delayed intracellular Ca^2+^ clearance, consistent with our earlier reports (Yang *et al*., 2006; Li & Ren, 2007; Li *et al*., 2007). Although Akt2 ablation failed to alter intracellular Ca^2+^ handling at young age, it reversed intracellular Ca^2+^ defects in aging, which may contribute to life span. Presence of intracellular Ca^2+^ mishandling with advanced aging is consolidated by reduced stress tolerance manifested as a steeper negative staircase in peak shortening‐frequency response. Our data revealed elevation of oxidized SERCA2a levels in aging hearts, which may account for, at least in part, intracellular Ca^2+^ mishandling and cardiac relaxation in senescent murine hearts. Moreover, our earlier report did not reveal any change in Akt1 and Akt3 isozymes with Akt2 ablation through adulthood (Xu *et al*., 2013), thus excluding possible compensatory regulation from Akt isozymes. It is noteworthy that deletion of Akt2 prompts onset and development of type 2 diabetes mellitus with time (Cho *et al*., 2001) although our current finding does not indicate that there is any diabetic phenotype at the young and old ages examined in our study.

Perhaps the most intriguing finding from our study is that Akt2 ablation prolonged life span and rescued against aging‐induced cardiac dysfunction despite more pronounced cardiac hypertrophy. The Akt upstream signaling such as insulin and IGF‐1 integrates environmental stimuli to regulate life span through Foxo transcription factors (phosphorylation in response to insulin/IGF‐1‐mediated Akt activation to expel Foxo from nucleus). Deletion of insulin and IGF‐1 promotes nuclear translocation of Foxo factors to enhance stress resistance and life span (Greer *et al*., 2009). Our observation of prolonged life span in Akt2^−/−^ mice is consistent with the notion of life span extension by inhibition of insulin, growth factors, and other Akt isozymes (Nojima *et al*., 2013; El‐Ami *et al*., 2014). The Akt2 knockout‐induced changes in cardiac mechanical function were coincided with improved mitochondrial integrity (UCP‐2, PGC‐1α, and aconitase activity), autophagy (Beclin‐1, LC3B, Atg7, and p62), and mitophagy (PINK1, Parkin, and BNIP3). Several theories may be proposed for Akt2 ablation‐elicited responses in aging. First, our result revealed that aging is associated with elevated plasma insulin and triglyceride along with a sustained Akt phosphorylation, suggesting hyperinsulinemia, chronic activation of Akt, onset, and development of insulin insensitivity in aging. However, the apparent dramatic reduction in AUC from IPGTT with aging does not seem to support presence of insulin resistance in our current experimental settings. The fact that Akt2 ablation rescued cardiac anomalies independent of the whole‐body metabolic status (levels of insulin and triglycerides, IPGTT) suggests a more cardiac effect rather than a secondary effect. Earlier findings from our group depicted dampened phosphorylation of the Akt‐negative regulator PTEN with aging (Hua *et al*., 2011), consistent with present observation of Akt activation in aging and the rationale of beneficial Akt2 ablation. Second, restored autophagy and mitophagy seem to play an important role for Akt2 ablation‐induced cardioprotection. Both Akt activation, a key molecule governing cardiac survival, autophagy, and mitochondrial function (Ronnebaum & Patterson, 2010; Wang *et al*., 2017), and aging have been shown to suppress autophagy (Cuervo *et al*., 2005; Zhang & Cuervo, 2008; Bjedov *et al*., 2010; Ronnebaum & Patterson, 2010; Taneike *et al*., 2010; Hua *et al*., 2011; Iida *et al*., 2011). Our results revealed that Akt2 ablation restored autophagy and mitophagy in aging hearts. Our *in vitro* findings further revealed that autophagy induction with rapamycin improved mitophagy and contractile function. It is likely that restored autophagy and mitophagy may be responsible for prolonged survival in Akt2 knockout mice, in line with the prolonged life span with autophagy induction (Harrison *et al*., 2009). Improved autophagy may improve diastolic function in senescent myocardium via preserved intracellular Ca^2+^ handling (Shinmura *et al*., 2011). Third, our findings suggested a role for mitochondrial autophagy and integrity in Akt2 ablation‐induced cardioprotection against aging. In our hands, Akt2 knockout alleviated aging‐induced changes in UCP‐2, mitochondrial biogenesis cofactor PGC‐1α, and mitochondrial aconitase activity along with suppressed mitophagy protein markers. Aging has been shown to trigger mitochondrial injury, leading to disrupted morphology, intracellular Ca^2+^ homeostasis, and contractile function (Tocchi *et al*., 2015). Mitochondrial homeostasis is maintained by protein quality control machineries such as fission/fusion, autophagy, and unfolded protein responses.

Our data suggested involvement of Sirt1‐dependent deacetylation of the transcriptional factor Foxo1 in cardiac aging, the effect of which was spared by Akt2 ablation. To the contrary, Akt2 ablation ameliorated aging‐induced Akt activation and Foxo1 phosphorylation. Sirt1 is known to activate Foxo1 by deacetylation (promoting nuclear localization) while Akt inhibits Foxo1 by phosphorylation (preventing nuclear translocation). Considering the downregulated Sirt1 levels with aging in WT and Akt2^−/−^ mice, the loss of Sirt1‐Foxo1‐mediated autophagy in aged mice may be compensated by Akt2 ablation‐offered disinhibition of Akt‐Foxo1‐mediated suppression of autophagy (Fig. S3, Supporting information). Finding from *in vitro* study suggested that Sirt1 activation using SRT1720 rescued impaired mitophagy and contractile dysfunction in aging, the effect of which was nullified by Akt activation using insulin or Parkin deficiency. Interestingly, SRT1720 failed to exert any notable mitophagy or contractile responses at young age. Loss of Parkin compromises mitochondrial quality by affecting mitochondrial biogenesis, bioenergetics, dynamics, transport, and turnover (Gautier *et al*., 2016). No additive action was noted between aging and Parkin deficiency, suggesting possible existence of Parkin defect with aging. We found that SRT1720‐mediated beneficial effect on mitophagy and cardiac function may be negated by insulin, suggesting a possible interplay between Sirt1 and Akt. It is plausible to speculate that Sirt1 activates Akt, resulting in phosphorylation of Foxo1 to inhibit Foxo activity in the presence of insulin (Akt activity). In the absence of Akt signaling, the lack of Akt‐driven Foxo1 phosphorylation (which relieves the inhibition on autophagy) with unchanged Sirt1‐mediated Foxo1 deacetylation should facilitate nuclear localization of Foxo1, resulting in improved stress resistance, endurance, and longevity. However, in the presence of Akt signaling such as in enriched nutrient environment, autophagy is expected to be suppressed by Akt‐Foxo1 phosphorylation. In addition, Sirt1 may directly activate Akt and IGF signaling to suppress autophagy and promote cellular senescence (Pillai *et al*., 2014), indicating a dual regulatory role for the longevity gene for autophagy and aging with various Akt status.

Experimental limitations: Although our data have provided causal relationships among cardiac morphology, function, mitochondrial integrity, and autophagy in aging, these results may not offer conclusive answers to the precise interplay among these factors in human aging process. Use of more unique models of autophagy or mitophagy should help to better understand the role of autophagy or mitophagy in the progression of cardiac aging and life span regulation.

In summary, our findings suggest that Akt2 seems play an essential role in the regulation of longevity, cardiac geometry, and function in aging. Our data favor the notion that increased Akt signaling and downregulated Sirt1 with advanced aging may underscore reduced autophagy and mitophagy in aging, indicating the therapeutic potentials for Akt and autophagy/mitophagy in the management of cardiac aging. Although our study sheds some light on the interaction of Akt‐Sirt1 signaling cascades on autophagy and cardiac homeostasis, the pathogenesis of cardiac dysfunction in aging, particularly in association with autophagy and mitochondria still deserves further investigation.

## Materials and methods

### Akt2 knockout (Akt2^−/−^) mice, Kaplan–Meier survival, and intraperitoneal glucose tolerance test (IPGTT)

All animal procedures were approved by the Animal Care and Use Committees at the University of Wyoming (Laramie, WY, USA) and Zhongshan Hospital Fudan University (Shanghai, China). Akt2 knockout (Akt2^−/−^) mice were obtained from Dr. Morris Birnbaum at the University of Pennsylvania (Philadelphia, PA, USA) and were characterized previously (Xu *et al*., 2013; Zhang *et al*., 2014). Both 3‐ to 4‐month‐old (denoted as ‘young’) and 24‐ to 26‐month‐old (as ‘old’) male wild‐type (WT) and Akt2^−/−^ mice were maintained on a 12‐h/12‐h light/dark cycle with free access to tap water and laboratory chow until experimentation. All mice used for life span analysis (the Kaplan–Meier survival curve and log‐rank test) were assigned to a longevity cohort at birth and were not used for any other tests. Prior to sacrifice, mice were fasted for 12 h and were then given an intraperitoneal (i.p.) injection of glucose (2 g kg^−1^ b.w.). Blood samples were drawn from tail veins immediately prior to the glucose challenge, as well as 15, 30, 60, and 120 mins thereafter. Blood glucose levels were determined using an Accu‐Chek III glucose analyzer. The area under the curve (AUC) was calculated using trapezoidal analyses for each adjacent time point and plasma glucose level. For *in vitro* study, cardiomyocytes from young (3–4 months old) or aged (24–26 months old) heterozygous Parkin (a cytosolic E3 ubiquitin ligase encoded by PARK2 gene) knockout mice were used (Gautier *et al*., 2016).

### Echocardiographic assessment

Cardiac geometry and function were evaluated in anesthetized (ketamine 80 mg kg^−1^ and xylazine 12 mg kg^−1^ i.p.) mice using the two‐dimensional‐guided M‐mode echocardiography (Phillips Sonos 5500) equipped with a 15–16‐MHz linear transducer (Phillips Medical Systems, Andover, MD, USA). Left ventricular anterior and posterior wall dimensions during diastole and systole were recorded from three consecutive cycles in M‐mode using methods adopted by the American Society of Echocardiography. Fractional shortening was calculated from LV EDD and ESD diameters using the equation (EDD − ESD)/EDD. Heart rate was averaged over 10 cardiac cycles (Xu *et al*., 2013).

### Isolation of mouse cardiomyocytes

Hearts were removed following anesthesia (ketamine 80 mg kg^−1^ and xylazine 12 mg kg^−1^ i.p.) and were mounted onto a temperature‐controlled (37 °C) Langendorff system. Following perfusion with a modified Tyrode solution (Ca^2+^ free) for 2 min, hearts were digested with a Ca^2+^‐free KHB buffer containing liberase blendzyme 4 (Hoffmann‐La Roche Inc., Indianapolis, IN, USA) for 20 min. The modified Tyrode solution (pH 7.4) contained the following (in mm): NaCl 135, KCl 4.0, MgCl_2_ 1.0, HEPES 10, NaH_2_PO_4_ 0.33, glucose 10, butanedione monoxime 10, and the solution was gassed with 5% CO_2_–95% O_2_. The digested heart was then removed from the cannula, and left ventricle was cut into small pieces in the modified Tyrode solution. Tissue pieces were gently agitated, and the pellet of cells was resuspended. Extracellular Ca^2+^ was added incrementally back to 1.20 mm over 30 min. A yield of at least 60–70% viable rod‐shaped cardiomyocytes with clear sarcomere striations was achieved. Only rod‐shaped myocytes with clear edges were selected for contractile and intracellular Ca^2+^ studies (Xu *et al*., 2013).

### Cell shortening/relengthening

Mechanical properties of cardiomyocytes were assessed using a softedge myocam ^®^ system (IonOptix Corporation, Milton, MA, USA) (Xu *et al*., 2013). In brief, cells were placed in a Warner chamber mounted on the stage of an inverted microscope (Olympus, IX‐70) and superfused (~1 mL min^−1^ at 25 °C) with a buffer containing (in mm): 131 NaCl, 4 KCl, 1 CaCl_2_, 1 MgCl_2_, 10 glucose, 10 HEPES, at pH 7.4. Cells were field‐stimulated at a frequency of 0.5 Hz (unless otherwise stated), 3‐msec duration, using a pair of platinum wires connected to a FHC stimulator (Brunswick, NE, USA). The myocyte being studied was displayed on the computer monitor using an IonOptix MyoCam camera. ionoptix softedge software was used to capture changes in cell length during shortening and relengthening. Cell shortening and relengthening were assessed using the following indices: peak shortening (PS) ‐ peak ventricular contractility, time‐to‐PS (TPS) ‐ contraction duration, and time‐to‐90% relengthening (TR_90_) ‐ cardiomyocyte relaxation duration, maximal velocities of shortening (+ dL/dt), and relengthening (− dL/dt) ‐ maximal velocities of ventricular pressure rise/fall. In the case of altering stimulus frequency from 0.1 to 5.0 Hz, steady‐state contraction of the myocyte was achieved (usually after the first 5–6 beats) before PS was recorded.

### Intracellular Ca^2+^ transient measurement

Myocytes were loaded with fura‐2/AM (0.5 μm) for 10 min, and fluorescence measurements were recorded with a dual‐excitation fluorescence photomultiplier system (IonOptix). Cardiomyocytes were placed on an Olympus IX‐70 inverted microscope and imaged through a Fluor × 40 oil objective. Cells were exposed to light emitted by a 75‐W lamp and passed through either a 360‐ or a 380‐nm filter, while being stimulated to contract at 0.5 Hz. Fluorescence emissions were detected between 480 and 520 nm by a photomultiplier tube after first illuminating the cells at 360 nm for 0.5 s then at 380 nm for the duration of the recording protocol (333 Hz sampling rate). The 360 nm excitation scan was repeated at the end of the protocol, and qualitative changes in intracellular Ca^2+^ concentration were inferred from the ratio of fura‐2 fluorescence intensity (FFI) at the two wavelengths (360/380). Fluorescence decay time was measured as an indication of intracellular Ca^2+^ clearing rate. Single exponential curve fit programs were used to derive intracellular Ca^2+^ decay constant (Doser *et al*., 2009).

### Histological examination

Following anesthesia, hearts were arrested in diastole with saturated KCl, excised, and fixed in 10% neutral‐buffered formalin at room temperature for 24 h. The specimen was processed through graded alcohols, cleared in xylenes, and embedded in paraffin. Serial sections were cut at 5 μm and deparaffinized slides were rinsed with PBS and incubated in 0.1 mg mL^−1^ fluorescein isothiocyanate (FITC)‐tagged lectin for 2 h. Thereafter, slides were washed with PBS three times, mounted with the aqueous mounting media. Cardiomyocyte cross‐sectional areas were digitalized using an Olympus BX‐51 microscope (Olympus America Inc., Melville, NY, USA). Frozen hearts were used for Masson trichrome staining. Serial sections were cut at 10 μm using a Leica, cryomicrotome (Model CM3050S, Leica Microsystems, Buffalo Grove, IL, USA) prior to fixation in 4% paraformaldehyde for 30 min. Following two washes with PBS, the sections were stained with a trichrome stain kit (Sigma‐Aldrich, St. Louis, MO, USA). Cardiomyocyte cross‐sectional area and fibrosis‐to‐total area ratio were measured from cardiomyocytes with clear myofiber outlines on a digital microscope (×400) using the imagej (version 1.34S) software (Doser *et al*., 2009).

### Aconitase activity

Mitochondrial aconitase, an iron–sulfur enzyme located in citric acid cycle, is readily damaged by oxidative stress via removal of an iron from [4Fe‐4S] cluster. Mitochondrial fractions prepared from whole‐heart homogenate were resuspended in 0.2 mm sodium citrate. Aconitase activity assay (Aconitase activity assay kit, Aconitase‐340 assay™, OxisResearch, Portland, OR) was performed according to manufacturer instructions with minor modifications. Briefly, mitochondrial sample (50 μL) was mixed in a 96‐well plate with 50 μL trisodium citrate (substrate) in Tris–HCl pH 7.4, 50 μL isocitrate dehydrogenase (enzyme) in Tris–HCl, and 50 μL NADP in Tris‐HCl. After incubating for 15 min at 37 °C, the absorbance was dynamically recorded at 340 nm every min for 5 min with a spectrophotometer. During the assay, citrate is isomerized by aconitase into isocitrate and eventually α‐ketoglutarate. The Aconitase‐340 assay™ measures NADPH formation, a product of the oxidation of isocitrate to α‐ketoglutarate. Tris–HCl buffer (pH 7.4) was served as blank (Hu *et al*., 2014).

### Separation of mitochondria

Ventricles were minced and homogenized by Polytron in the ice‐cold MSE buffer [220 mm mannitol, 70 mm sucrose, 2 mm EGTA, 5 mm 3‐(4‐morpholino) propane sulfonic acid (MOPS), pH 7.4, 0.2% bovine serum albumin (BSA) and a protease inhibitor cocktail containing 4‐(2‐aminoethyl) benzenesulfonyl fluoride (AEBSF), E‐64, bestatin, leupeptin, aprotinin, and EDTA obtained from Sigma Chemicals (St. Louis, MO, USA)]. The homogenates were centrifuged for 10 min at 600×*g* to remove unbroken tissue and nuclei, and supernatants were centrifuged for 10 min at 3000×*g* to pellet mitochondria. The mitochondrial pellet was dissolved in the protein lysis buffer and centrifuged at 10 000×*g* for 30 min at 4 °C to make a soluble protein. Fifty micrograms of mitochondrial protein was separated by 15% sodium dodecyl sulfate–polyacrylamide gel electrophoresis (SDS‐PAGE) for Western blot analysis (Hu *et al*., 2014).

### Western blot analysis

Murine heart tissues were homogenized and sonicated in a lysis buffer containing 20 mm Tris (pH 7.4), 150 mm NaCl, 1 mm EDTA, 1 mm EGTA, 1% Triton, 0.1% sodium dodecyl sulfate (SDS), and a protease inhibitor cocktail (Thermo Scientific). The protein concentration of the supernatant was evaluated using Protein Assay Reagent (Bio‐Rad, Hercules, CA, USA). Equal amounts (50 μg protein/lane) of the protein from the tissue extraction, or prestained molecular weight markers (Gibco‐BRL, Gaithersburg, MD, USA) were separated on 10% or 15% SDS–polyacrylamide gels in a minigel apparatus (Mini‐PROTEAN II; Bio‐Rad). Protein levels of Sirt1, LC3B, Beclin‐1, Atg7, p62, PINK1, Parkin, BNIP3, UCP‐2, PGC‐1α, Akt, phosphorylated Akt (pAkt, Ser^473^), Foxo1, phosphorylated Foxo1 (pFoxo1, Ser^256^), and acetylated Foxo1 (acFoxo1) were examined using standard Western immunoblotting. All antibodies were obtained from Cell Signaling Technology (Beverly, MA, USA) except p62 antibody [PROGEN Biotechnik (Heidelberg, Germany)]. Membranes were probed with respective antibodies using α‐tubulin as the loading control. To detect the expression of cellular triton‐insoluble p62, cellular proteins were separated into detergent‐soluble and detergent‐insoluble fractions with the 2% Triton X‐100 buffer (50 mm Tris (pH 8.0), 150 mm NaCl, 1 mm EDTA, 10% glycerol, 2% Triton X‐100, protein inhibitor cocktail). Buffer with 1% SDS was used to solve the insoluble fractions. The membranes were incubated with horseradish peroxidase (HRP)‐coupled secondary antibodies. Following immunoblotting, the film was scanned and detected using a Bio‐Rad Calibrated Densitometer, and the intensity of the immunoblot bands was normalized to that of GAPDH or COX‐IV (Doser *et al*., 2009).

### Data analyses

Data are expressed as mean ± SEM. Statistical significance (*P* < 0.05) was estimated using one‐way analysis of variation (ANOVA) or repeated measures of ANOVA (for Fig. 1B) followed by a Tukey's test for *post hoc* analyses. Log‐rank test was used for Kaplan–Meier curve.

## Funding

This work was supported in part by grants from the National Institute of Health/National Institute of Aging (R03 AG21324), and the National Natural Science Foundation of China (81522004, 81570225, 81370195, 81521001).

## Author contributions

JR, LY, and YZ designed the experiments, conducted the study, and prepared the manuscript; JR, LY, XX, AFC, WG conducted the study; LZ, WG, and JY reviewed the manuscript and contributed to the discussion.

## Conflict of interest

None declared.

## Supporting information


**Fig. S1** Representative gel blots depicting levels of Akt2 from male young or old WT and Akt2^−/−^ mice (GAPDH used as loading control).
**Fig. S2** Role of autophagy in Akt2 ablation‐induced beneficial response against cardiac aging.
**Fig. S3** Schematic diagram depicting the role of Akt, Sirt1 and Foxo1 in aging‐induced changes in autophagy, mitochondrial integrity and cardiac function.
