# Correction to: Akt2 ablation prolongs life span and improves myocardial contractile function with adaptive cardiac remodeling: role of Sirt1‐mediated autophagy regulation

**DOI:** 10.1111/acel.14119

**Published:** 2024-03-12

Ren J, Yang L, Zhu L, Xu X, Ceylan AF, Guo W, Yang J, Zhang Y. Aging Cell. 2017;16(5):976‐987. doi: https://doi.org/10.1111/acel.12616. Epub 2017 Jul 5.

The authors of the abovementioned paper noticed an error in Figure 1 as reported. During data analysis of lectin staining, several data points were mistakenly entered twice causing erroneously reported sample size, mean, and SEM. We have corrected this mistake and summary bar graph (and updated representative image to better match with mean value of cardiomyocyte area). Conclusion was not affected by this error. The authors sincerely regret this error and wish to thank the editorial office and publisher for this correction.

**FIGURE 1:**
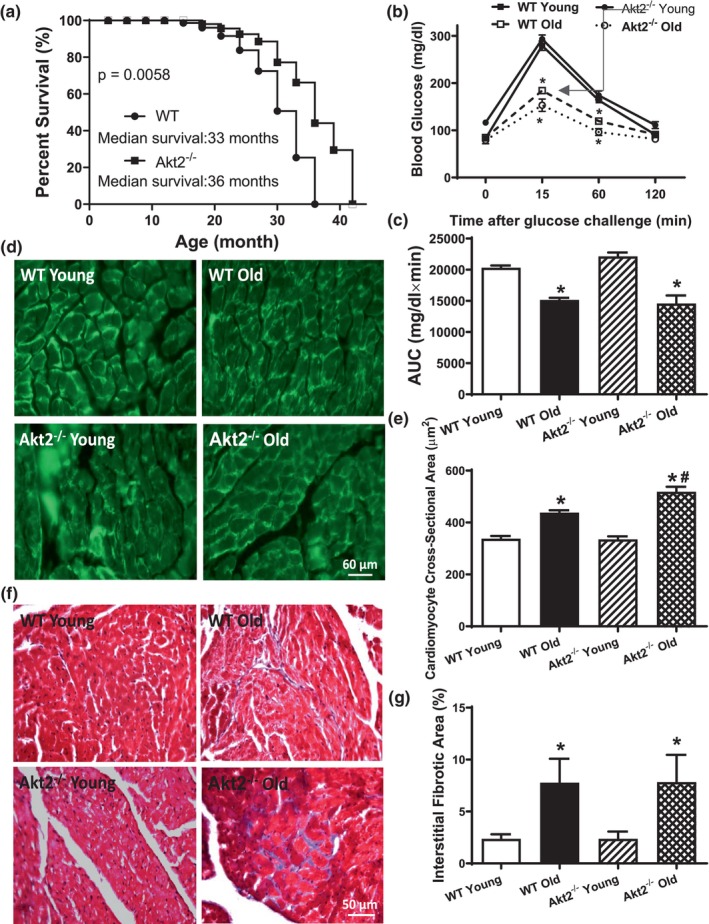
Cumulative survival curve, intraperitoneal glucose tolerance test (IPGTT, 2 g/kg), area underneath curve (AUC), and myocardial morphology in male WT and Akt2 knockout (Akt2^−/−^) mice at young (3–4 months) and old (24–26 months) ages. (a) Kaplan–Meier cumulative survival rate plotted against age in months in WT and Akt2^−/−^ mice. The log rank test was performed to compare the two curves (*p* = 0.0058). (b) Plasma glucose levels within 120 min following IPGTT challenge; (c) AUC; (d) Representative FITC‐conjugated lectin staining depicting the transverse sections of left ventricular myocardium (×400); (e) Quantitative analysis of cardiomyocyte cross‐sectional area; (f) Representative Masson trichrome staining depicting interstitial fibrosis; and (g) Quantitative analysis of fibrotic area (Masson's trichrome stained area in light blue color normalized to the total myocardial area). Mean ± SEM, *n* = 31 mice per group for panel a; 6–7 mice per group for panels b and c; 15–17 fields from 5 mice per group for panels d and e; and 8–9 fields from 5 mice per group for panels f and g, *p < 0.05 versus respective WT young, ^#^ *p* < 0.05 versus WT old group.

We apologize for this error.

