# Supplementary material for: Akt2 ablation prolongs life span and improves myocardial contractile function with adaptive cardiac remodeling: role of Sirt1‐mediated autophagy regulation
Source: Aging Cell. 2017 Jul 5;16(5):976–87. doi: 10.1111/acel.12616 (PMC5595687; doi:10.1111/acel.12616)
Supplement: Supplementary file 1 — Fig. S1 Representative gel blots depicting levels of Akt2 from male young or old WT and Akt2−/− mice (GAPDH used as loading control). Fig. S2 Role of autophagy in Akt2 ablation‐induced beneficial response against cardiac aging. Fig. S3 Schematic diagram depicting the role of Akt, Sirt1 and Foxo1 in aging‐induced changes in autophagy, mitochondrial integrity and cardiac function. [file ACEL-16-976-s001.doc]

**Supplemental Materials**

**Akt2 Ablation Prolongs Lifespan and Improves Myocardial Contractile Function with Adaptive Cardiac Remodeling: Role of Sirt1-Mediated Autophagy Regulation**

**Jun Ren*^1,2^, Lifang Yang*^2,4^, Li Zhu^1^, Xihui Xu^2^, Asli F. Ceylan^2^, Wei Guo^3^, Jian Yang^5^ and Yingmei Zhang^1,2^**

**^1^Department of Cardiology and Shanghai Institute of Cardiovascular Diseases, Zhongshan Hospital, Fudan University, Shanghai 200032, China; ^2^Center for Cardiovascular Research and Alternative Medicine, School of Pharmacy, ^3^Department of Animal Sciences, University of Wyoming, Laramie, WY 82071 USA; ^4^Department of Anesthesiology, Xi’an Children Hospital, Xi’an 710003; and ^5^Department of Cardiovascular Surgery, Xijing Hospital, Fourth Military Medical University, Xi’an, 710032, China**

# Running title: Akt2 ablation, autophagy and cardiac aging

# *: Equal contribution

**Correspondence to:**

**Dr. Jun Ren or Dr. Yingmei Zhang**

**University of Wyoming College of Health Sciences, Laramie, WY 82071, USA**

**Tel: 307-766-6131; Fax: 307-766-2953; E-mail:** [jren@uwyo.edu](mailto:jren@medicine.nodak.edu); [zhangym197951@126.com](mailto:zhangym197951@126.com)

Supplemental Fig. 1: Representative gel blots depicting levels of Akt2 from male young or old WT and Akt2^-/-^ mice (GAPDH used as loading control).

Ren et al., Fig. 7

**A.**

**C.**

**E.**

**B.**

**D.**

**F.**

Supplemental Fig. 2: Role of autophagy in Akt2 ablation-induced beneficial response against cardiac aging. Cardiomyocytes from young and old Akt2^-/-^ mice were co-incubated with the autophagy inhibitor 3-methyladenine (3-MA, 3 mM) for 4 hrs prior to mechanical recording. Cardiomyocytes from young (4-mo) and old (24-mo) WT mice were used as controls. A: Resting cell length; B: Peak shortening (% of resting cell length); C: Maximal velocity of shortening (+ dL/dt); D: Maximal velocity of relengthening (- dL/dt); E: Time-to-peak shortening (TPS); and F: Time-to-90% relengthening (TR_90_). Mean ± SEM, n = 38 -39 cells per group, *p < 0.05 *vs.* WT-Young group; #p < 0.05 *vs.* WT-Old group, † p < 0.05 *vs.* the corresponding 3-MA absent group

Ren et al., Fig. 7

Supplemental Fig. 3:  Schematic diagram depicting the role of Akt, Sirt1 and Foxo1 in aging-induced changes in autophagy, mitochondrial integrity and cardiac function. Aging promotes Akt phosphorylation and downregulates Sirt1. Sirt1 promotes Akt phosphorylation en route to Foxo1 phosphorylation and stimulates Foxo1 deacetylation, while exert opposite (offsetting) effects on autophagy and mitophagy. Akt2 ablation inhibits Foxo1 phosphorylation thus favoring autophagy/mitophagy through Foxo1 deacetylation-mediated mechanism, leading to improved mitochondrial integrity.

Ren et al., Fig. 7
